# Acute‐onset chronic inflammatory demyelinating polyneuropathy with cranial nerves and respiratory tract involvement: A case report

**DOI:** 10.1002/ccr3.3087

**Published:** 2020-07-24

**Authors:** Malek Mansour, Asma Ouerdiene, Ines Bedoui, Amel Kacem, Jamel Zaouali, Ridha Mrissa

**Affiliations:** ^1^ Department of Neurology Military Hospital of Instruction of Tunis Tunis Tunisia; ^2^ Department of Medicine Regional Hospital of Jendouba Jendouba Tunisia

**Keywords:** acute‐onset chronic inflammatory demyelinating polyneuropathy, Bulbar and respiratory tract involvement, chronic inflammatory demyelinating polyneuropathy, Guillain‐Barré syndrome

## Abstract

Sixteen percent of chronic inflammatory demyelinating polyneuropathy (CIDP) patients may present acutely like acute idiopathic demyelinating polyneuropathy (AIDP) the demyelinating form of GBS, developing in <8 weeks 2. This entity is classified as acute‐onset CIDP (A‐CIDP) which presents overlapping clinical and electrophysiological findings with GBS during early stages of disease, but followed with a chronic course beyond 2 months. Also, those who have three or more treatment‐related fluctuations (TRF) are included under this term.

Distinguishing between acute‐onset chronic inflammatory demyelinating polyneuropathy (A‐CIDP) and acute idiopathic demyelinating polyneuropathy (AIDP) may be difficult during early stages but is crucial in order to guide treatment strategies without delay. These two forms share some overlapping clinical and electrophysiological findings, including some severe clinical features such as cranial nerve and respiratory tract involvement making the diagnosis of A‐CIDP more difficult.

## Graphical abstract

Sixteen percent of chronic inflammatory demyelinating polyneuropathy (CIDP) patients may present acutely like acute idiopathic demyelinating polyneuropathy (AIDP) the demyelinating form of GBS, developing in <8 weeks (Ruts et al *Neurology*, **74**, 2010 and 1680). This entity is classified as acute‐onset CIDP (A‐CIDP) which presents overlapping clinical and electrophysiological findings with GBS during early stages of disease, but followed with a chronic course beyond 2 months. Also, those who have three or more treatment‐related fluctuations (TRF) are included under this term.

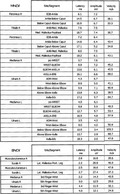

## 1 INTRODUCTION

Chronic inflammatory demyelinating polyneuropathy (CIDP) is an acquired autoimmune inflammatory polyneuropathy characterized by a progression over at least 2 months with a progressive or relapsing course. However, it has been reported that a significant number of patients with CIDP (16%) may present with an acute clinical onset reaching a nadir within the first 8 weeks and a subsequent chronic progression sharing similar clinical and electrophysiological findings, during early stages of disease, with the AIDP, the demyelinating variant of Guillain‐Barré syndrome (GBS). These patients are classified as acute‐onset CIDP (A‐CIDP)^2^. Distinguishing AIDP from A‐CIDP is crucial in order to guide treatment strategies and to predict long‐term prognosis. Here, we discuss the main clinical and evolutive particularities of A‐CIDP through a case of a 59‐year‐old male diagnosed with A‐CIDP.

## 2 CASE REPORT

**FIGURE 1:**
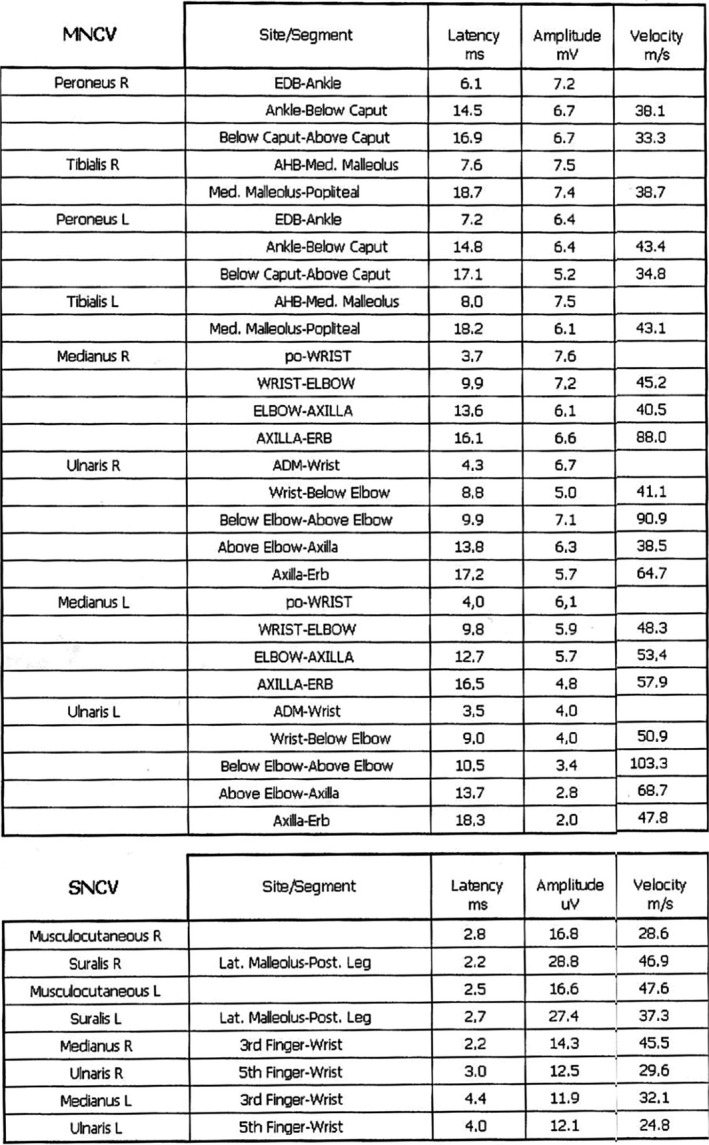
Resuming table of motor and sensory nerve conduction findings

**FIGURE 2:**
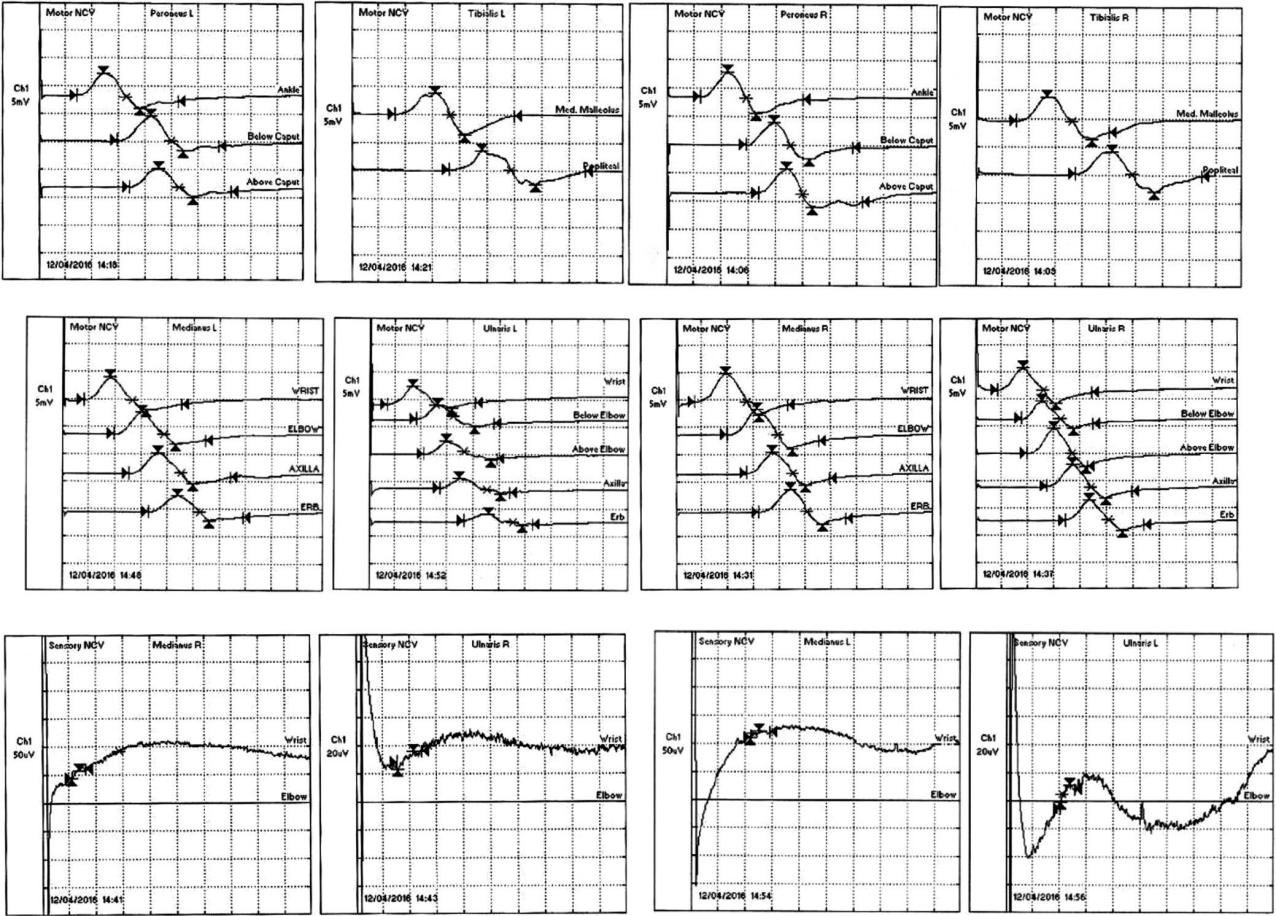
Latency and amplitude study

**FIGURE 3:**
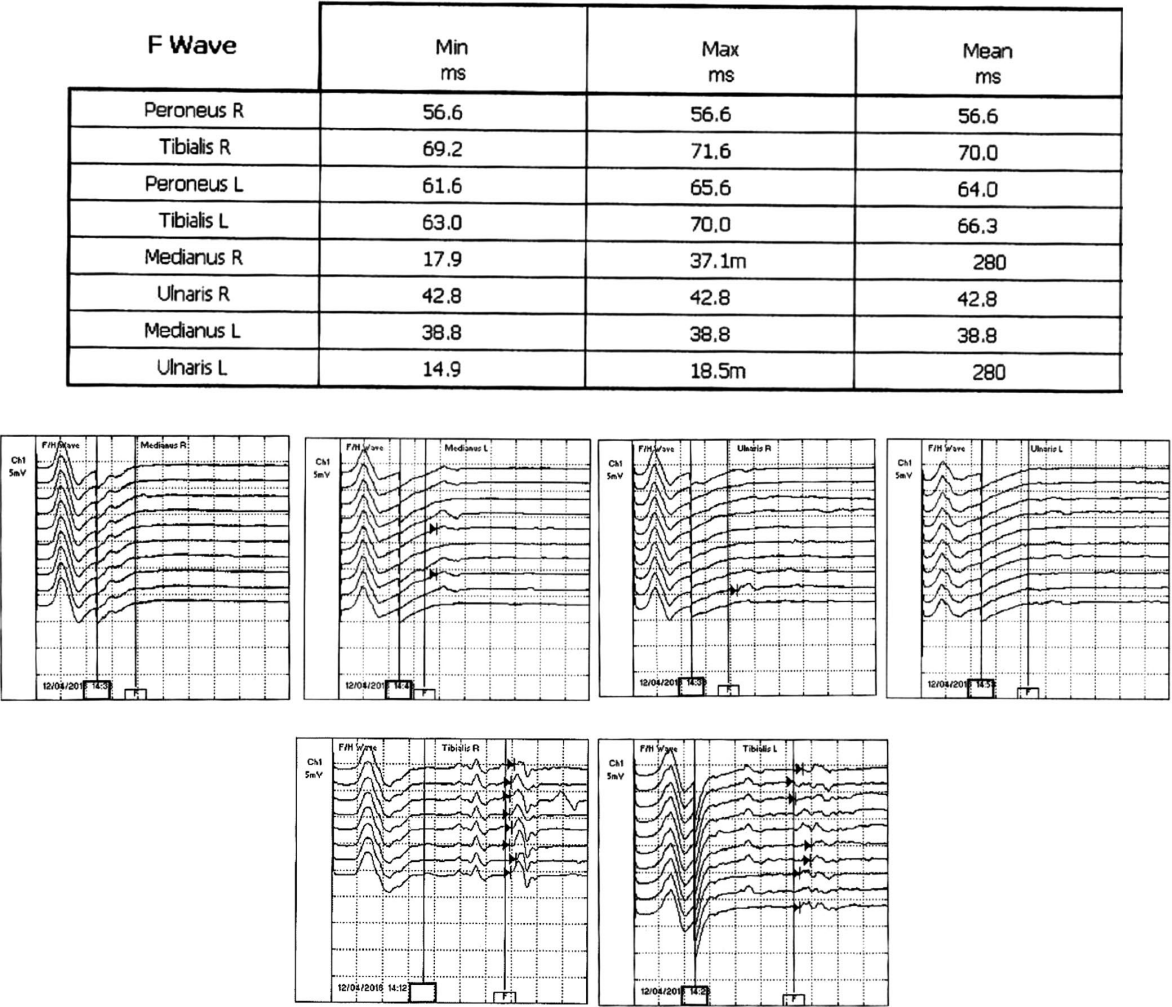
F‐wave study

A 59‐year‐old man, without any particular medical history, presented at our department of neurology at the military hospital with a progressive clinical picture, 10 days following a flu, made of initial ascending paresthesia attending rapidly the upper limbs in <24 hours followed after 2 days by weakness in the lower then the upper limbs, and a discrete dysphagia with frequent choking to liquids. He was admitted to our hospital 8 days after the beginning of the symptoms on 11 April 2016. During hospitalization, he developed 3 days after his admission, respiratory weakness with worsening of the dysphagia, and facial diplegia on the 14th day of evolution. No autonomic dysfunction was noted. Physical examination showed flaccid tetraplegia graded 3 in upper limbs and 2 in lower limbs according to MRC scale, with absence of all reflexes, hypoesthesia in glove and stocking distribution, a positive Romberg sign, and facial diplegia. The EMG (Figures 1, 2 and 3) showed sensory and motor demyelinating polyradiculoneuropathy with decreased motor conduction velocities, prolonged distal latencies of ulnaris, medianus, tibilais, and peroneus, prolonged F‐wave latencies in the four limbs, with decreased sensory conduction velocities of the right musculocutaneous and left ulnaris. CSF analysis highlighted albumino‐cytological dissociation with 0 white cell and CSF protein level at 0.7 mg/dL. The patient was initially diagnosed as GBS and received an immunoglobulin therapy. His symptoms improved then progressively, and he was discharged on 25 April 2016, after the total regression of the respiratory signs and the improvement of the dysphagia, facial diplegia, and muscle weakness which was graded 4+ at his discharge. Two weeks later, he presented with worsening of the dysphagia and a sensory ataxia. He was readmitted to our department on 10 May 2016. Physical examination showed sensory ataxia, positive Romberg sign, loss of deep sensation at right, absence of all reflexes, with the same muscle strength grade 4 at his discharge, a weak nausea reflex, and a discrete facial diplegia. This was considered as a treatment‐related fluctuations (TRF), and the patient received a second immunoglobulin therapy with a complete regression of the dysphagia and the sensory ataxia. After his discharge, he continued to improve with a clear regression of symptoms and recovery of all reflexes, only a discrete proximal muscle weakness persisted with few paresthesia of extremities which responded well to pregabalin. One year later, on April 2017, the patient manifested recurrent symptoms with flaccid tetraplegia graded 4, absence of all reflexes, and a positive Romberg sign noted at the physical examination. He was then differentially diagnosed with A‐CIDP according to Ruts et al criteria.^2^ He started taking methylprednisolone daily with a total recovery. The evolution was marked by steroid dependence, leading to the association of an immunosuppressive agent (azathioprine) with stability of the disease. He only kept a discrete proximal muscle weakness in lower limbs.

## 3 DISCUSSION

Although CIDP is defined as a chronic progressive or relapsing condition that develops over at least 2 months, studies have shown that up to 16% of CIDP patients may present acutely like AIDP the demyelinating form of GBS, developing in <8 weeks.^2^ This entity is classified as acute‐onset CIDP (A‐CIDP) which presents overlapping clinical and electrophysiological findings with GBS during early stages of disease, but followed with a chronic course beyond 2 months. Also, those who have three or more treatment‐related fluctuations (TRF), during which secondary deterioration lasting <8 weeks is observed after treatment,^4^ were included under this term according to Ruts et al.^2^ Many studies^1^, ^2^, ^4^, ^5^ were conducted to find these distinctive clinical features between patients with AIDP and A‐CIDP to help predict clinical behavior of patients with A‐CIDP and guide treatment strategy and prognosis. All these studies demonstrated that A‐CIDP was more frequent in men. The mean age at onset of symptoms was 43 years in Dionne et al study,^1^ 47 years in Ruts et al,^2^ and 55.5 years in Alessandro et al study^4^ closer to our patient who was 59 years old at the onset of symptoms. The infectious prodrome presented by our patient was rarely correlated with A‐CIDP in the Dionne et al study but nonsignificant difference was founded in Ruts et al and Alessandro et al studies. A‐CIDP patients in Dionne et al and Alessandro et al studies were significantly more likely to have prominent sensory signs with proprioceptive disturbances and sensory ataxia, orientating to A‐CIDP rather than GBS, which was noted in our patient. Overmore, they were significantly less likely to have facial or bulbar weakness (defined as dysphagia, dysarthria), and respiratory tract involvement, all found in our patient, with only one patient out of 8 (13%) having facial weakness in Ruts et al study, three patients out of 15 (20%) in Dionne et al study, and four out of 14 (29%) with only one patient having facial diplegia in Alessandro et al study. Bulbar weakness was described in three patients (20%) in Dionne et al, in only one patient (7%) in Alessandro et al, and in no patient in Ruts et al study. Only two patients (25%) presented respiratory muscle weakness in Ruts et al study, and three patients (20%) in Dionne et al study. However, in this case, our patient had facial weakness with facial diplegia, bulbar involvement with dysphagia, and mild respiratory muscle weakness. Electrophysiological studies are not useful to distinguish A‐CIDP from AIDP in the early stages, but they may be helpful in more advanced stages. In fact, only 25% of patients with AIDP meet criteria for demyelinating neuropathy after 26‐35 weeks,^5^ whereas the long‐term demyelination is constant in patients with A‐CIDP.^3^

Early recognition of A‐CIDP in patients with apparent GBS is clinically difficult, but important. In fact, distinguishing AIDP from A‐CIDP is crucial because treatment strategies and long‐term prognosis are different. In fact, AIDP will respond to a single dose of gammaglobulin (IVIg) or to plasmapheresis, with only 8 to 16% patients presenting treatment‐related fluctuations (TRF), during which secondary deterioration lasting <8 weeks is observed after treatment by immunoglobulin.^4^, ^6^ This is relied to the drug‐related pharmacokinetics which has a half‐life of 21 days, requiring then a repeated immunoglobulin therapy or plasma exchanges. While A‐CIDP similarly to CIDP requires long‐term immunosuppressive treatment in most cases, including corticosteroids.^4^

The aim of this study is to highlight some clinical and evolutive particularities of A‐CIDP especially the respiratory tract involvement and bulbar signs which are rarely described in this particular subtype of CIDP making the diagnosis more difficult in early stages.

## 4 CONCLUSION

Acute‐onset chronic inflammatory demyelinating polyneuropathy is a particular CIDP subtype. It presents 16% of CIDP and is characterized by an acute‐onset resembling GBS at early stages, developing in <8 weeks but followed with a chronic course beyond 2 months. Cranial nerves and respiratory tract involvement in this form are rare but have been described. Further studies need to be conducted in order to better characterize this different single entity.

## CONFLICT OF INTEREST

None declared.

## AUTHOR CONTRIBUTION

MM: Conceptualization, Resources, Investigation, Methodology, Validation, Visualization, Supervision. AO: Investigation, Formal analysis, Data curation, Writing ‐ review & editing, original draft, Visualization. IB: Investigation, Formal analysis, Visualization. AK: Formal analysis, Visualization. JZ: Validation, Supervision, Project administration. RM: Validation, Supervision, Project administration.
